# Feasibility of Cervical Epidural Anesthesia for Breast Cancer Surgery

**DOI:** 10.1155/2017/7024924

**Published:** 2017-07-18

**Authors:** Manuel Wenk, Christina Massoth, Daniel M. Pöpping, Michael Möllmann

**Affiliations:** ^1^Department of Anesthesiology, Intensive Care and Pain Medicine, University Hospital Muenster, Muenster, Germany; ^2^Department of Anesthesiology and Intensive Care, St. Franziskus Hospital, Muenster, Germany

## Abstract

**Background:**

Effects of perioperative cervical level neuraxial blocks on the dissemination of cancer metastases have become a matter of substantial interest. However, experience with these catheters has been limited and data on feasibility and efficacy is sparse.

**Methods:**

Data from 39 patients scheduled to undergo breast cancer surgery while awake with a cervical epidural alone was retrospectively analyzed.

**Results:**

In 26 patients (66,7%, 95% CI 51,7–81,7) the cervical epidural catheter was sufficient for surgery. In one patient (2.6%, 95% CI 0–7.6) identification of the epidural space was not possible. Four patients (10.3%, 95% CI 0,7–19,9) had an insufficient sensory block. Seven patients (17.9%, 95% CI 5,7–30,1) had a partially insufficient sensory block. Rates of failed epidural blocks were not significantly different between different insertion levels. 21 patients (80.8%, 95% CI 65,4–96,1) developed hypotension and required an intravenous vasopressor. One patient developed nausea. In one patient the dura was accidentally punctured. No neurological damage was observed. No other major complications were observed.

**Discussion:**

Epidural punctures in the cervical region are feasible but do bear potential for major complications. Anesthesiologists should familiarize themselves with high epidural block techniques.

## 1. Introduction

Cancer remains one of the leading causes of death in developed countries. While lung cancer is predominant in men (17%), women suffer most frequently from breast cancer (23%) [1]. While the primary tumor can be removed in almost all cases, the dilemma is that 90% of all cancer patients die from metastatic disease [2]. Over the last couple of years the role of a perioperatively compromised immune system leading to easier dissemination of tumor cells and accelerated growth of micrometastases has been extensively discussed [3–5]. It appears that reduced perioperative stress might transfer into a better immune response of the patient; hence anesthetic techniques per se have become a significant field of research [6–9]. Particularly the effects of epidural anesthesia have been the center of attention since neuraxial blocks generally provide excellent analgesia and potentially reduce perioperative stress resulting in an overall reduction of the requirement for other anesthetic and analgesic agents in the postoperative period [10–12]. However, despite intensified research, clinical evidence for a potentially protective effect of regional anesthetic and analgesic techniques with respect to cancer recurrence and the incidence of metastases after tumor surgery is still inconclusive [13].

Consequently, with no effect of regional anesthetic techniques known to promote the growth of tumor or metastases, it appears that these techniques should be widely employed whenever possible. However, particularly in the case of breast cancer surgery, a sufficient epidural technique requires the insertion of a cervical epidural catheter at the C6/C7/T1 level. In stark contrast to the high volume of breast cancer surgery performed in many hospitals stands the relatively rarely performed anesthetic technique of cervical epidural catheter insertion, which both can be challenging for the anesthetist and involves potentially devastating sequelae for the patient. Traditionally, cervical epidural catheters have only been used in selected centers performing cardiac or other high risk surgery; hence the familiarity of anesthetists with this technique is not necessarily ubiquitous. The available data on feasibility and efficacy of cervical epidural anesthesia is sparse, consisting mainly of case reports reporting complications and a few studies from the field of pain medicine [14, 15].

In this retrospective study we sought to verify the feasibility in terms of success/failure rates and investigated complications in a series of patients undergoing breast cancer surgery while awake with a cervical epidural catheter.

## 2. Methods and Materials

After approval from the Ethics Committee of the University of Muenster and the Medical Board of North-Rhine Westphalia (protocol number 2012415fN), data from 39 consecutive women planning to undergo breast cancer surgery while awake with only a cervical epidural catheter and no general anesthesia was retrospectively analyzed.

Briefly, patients with a normal coagulation profile (INR < 1.4, aPTT < 41 sec, and platelets > 80.000 per microliter of blood) were connected to standardized anesthetic monitoring, including electrocardiogram, noninvasive blood pressure with a 3 min cycle period, and peripheral oxygen saturation. An invasive blood pressure monitoring was used based on the patient's individual risk factors. All patients received a standardized cervical epidural catheter insertion by the same anesthesiologist preoperatively. Patients were placed in the lateral position with their heads anteflexed. The seventh cervical vertebra was marked as an orientation landmark and the area was thoroughly cleaned and disinfected. Operator was using sterile gloves, gown, and face mask for all procedures. The area was covered with sterile draping and the skin and subcutaneous tissue were infiltrated with 2–5 ml prilocaine 1% (Xylonest 1%, Astra Zeneca, Germany). The epidural space was punctured at the vertebrae level C6/C7 or C7/T1 or T1/T2 with a standard 18-gauge Tuohy needle (B. Braun, Melsungen, Germany) using a midline approach with a loss of resistance to air technique. An epidural catheter was then inserted via the Tuohy needle and advanced cranially in the epidural space for 2 cm. The Tuohy needle was then removed and the catheter was fixated and covered with sterile dressings. A bolus of 10 ml ropivacaine 0.75% (Astra Zeneca, Hamburg, Germany) was administered. Patients were then turned supine and after 7–10 minutes a further 3–5 ml ropivacaine 0.75% was given. Sufentanil (Actavis, Munich, Germany) was available to be used for epidural application at the anesthetist's discretion. Surgery was then started following a 20- to 30-minute wait. Postoperatively, the catheter was removed and after fading of the effects of the epidural anesthesia, patients were discharged from recovery and followed up two hourly for 12 postoperative hours.

Data with regard to patient and surgical characteristics, operator experience, site of insertion, and complications as well as the use of cardiovascular drugs was collected. Complications were divided into none, minor, major, or failed attempt. The following complications were defined as minor ones: inability to reach the epidural space, multiple attempts, change of insertion site or a second operator, bleeding from the epidural needle or blood in the epidural catheter, and difficulties advancing the epidural catheter as well as nausea, vomiting, or vertigo.

Dural puncture, nerve injury or any other neurological complications, dyspnea or difficulty in breathing as well as significant hemodynamic depression (systolic blood pressure < 90 mmHg and/or heart rate < 50 or >120 beats per minute after injection of local anesthetic), or any other significant events were considered major complications.

All data was entered into a customized database. Statistical analysis was performed using SPSS Statistics 18.0 (SPSS Inc., Chicago, IL). Categorical variables are expressed as frequency and percentage, whereas continuous variables are represented as means with standard deviation. The nonparametric patients' baseline characteristics were assessed using the Kruskal-Wallis test. Differences were considered statistically significant at *p* < 0.05.

## 3. Results

39 patients aged 72 years (range: 29–92) underwent breast cancer surgery while awake using a cervical epidural catheter as the sole method of anesthesia. Demographic and surgery-related data of the patient population is shown in Table 1.

In 26 patients (66,7%, 95% CI 51,7–81,7) the cervical epidural catheter was sufficient as the sole method of anesthesia to perform breast cancer surgery.

In one patient (2.6%, 95% CI 0–7.6) identification of the epidural space was not possible and the procedure was aborted. Four patients (10.3%, 95% CI 0,7–19,9) had an insufficient sensory block leading to a conversion of anesthetic method from cervical epidural catheter to general anesthesia. Seven patients (17.9%, 95% CI 5,7–30,1) had a partially insufficient sensory block and anesthesia had to be supplemented with intermittent IV application of S-ketamine and/or midazolam.

Rates of total and partially insufficient epidural blocks were not significantly different between the three catheter insertion levels (C6/7 versus C7/T1 versus T1/T2).

To investigate the effect of a possible operator learning curve, the first 20 cervical epidural punctures were compared with the following 19 punctures. No significant changes in success/failure rates were observed.

21 of the 26 patients (80.8%, 95% CI 65,4–96,1) with a sufficient cervical epidural received at least one dose of an IV vasopressor after epidural puncture due to hypotension. 16 patients received fractionated doses of 20 mg cafedrine and 1 mg theodrenaline (Akrinor®; AWD.pharma, Dresden, Germany), three patients received intermittent doses of 5–10 *μ*g nor-epinephrine (Arterenol®, Sanofi-Aventis, Frankfurt, Germany), and two patients received a combination of the two drugs. Reflex bradycardia was observed but no intervention was necessary. No patient developed a significant isolated bradycardia.

One patient received an epidural injection of 5 *μ*g sufentanil in addition to the local anesthetic and rapidly developed severe nausea and vomiting. No other patient received any additional opioids. One patient developed late (>6 hrs) postoperative nausea and vomiting.

In one patient the dura was accidentally punctured at the C7/T1 level and the procedure was aborted. No neurological damage or any other sequelae were observed.

No other major complications were observed; particularly, no nerve or other neurological damage was seen during the observation period. No patient suffered from respiratory complications such as dyspnea or apnea.

## 4. Discussion

Our study has shown that cervical epidural anesthesia is a feasible technique with an acceptable success rate that offers sufficient and profound analgesia for breast cancer surgery while patients are awake.

With increasing evidence that neuraxial blocks such as epidural or paravertebral anesthesia may influence the degree of dissemination of metastatic disease [3, 6, 7, 12, 16, 17] anesthesiologists should no longer consider these techniques as rare methods performed in highly specialized centers. Even if the scientific evidence regarding the precise role of these techniques on cancer recurrence is still questioned by some experts [18], there is no known effect where these methods would in any way promote the spread of cancer cells. Hence the potential benefits in reducing postoperative pain alone appear to outweigh the risks of the method; hence anesthesiologists today find themselves in a situation where they need to discuss why they are not liberally employing these techniques. One obvious reason, particularly in breast cancer surgery, is the requirement of a high cervical level block which can be technically challenging and usually only few anesthesiologists would have had previous exposure to these techniques since they are usually restricted to selected centers. With breast cancer surgery being performed in many hospitals this gives rise to a significant discrepancy between the demand for cervical epidurals and the ability of the staff to perform these techniques.

In our study we were able to site a cervical epidural correctly in 67% of all patients. Although a failure rate of 32% may appear high at first glance it should be noted that this reflects epidural catheters that were sufficient to perform a surgical procedure without any further anesthesia necessary. 28% of patients had a partial block that needed either conversion to general anesthesia or supplementation with other analgesics/hypnotics. As such, the complete failure rate was rather low and since epidural blocks and general anesthesia are usually performed in combination it can be speculated that many partially working epidurals would have been considered fully functioning. The combined partial and total failure rates of epidural catheters are comparable to published data. Studies as well as a recent review have highlighted failure rates as high as 32% for thoracic and 27% for lumbar epidural catheters, which not only were total failures but also involved partial failures [19, 20]. This raises the important but yet unanswered question whether full or partial blocks do have different effects in their potential influence on intraoperative stress levels as well as on the spread of cancer cells during surgery. Differentiating between a fully and a partially functional neuraxial block may also be an important limitation for all prospective clinical trials currently underway.

Neuraxial anesthesia for breast surgery remains the gold standard in terms of providing postoperative analgesia with some studies even suggesting a reduction in the incidence of postoperative persistent pain, though the quantity of available evidence remains severely limited [21]. As an alternative to cervical epidural anesthesia, thoracic paravertebral block (TPVB) appears like a viable option that has shown some beneficial effects on postoperative pain intensity, perioperative opioid consumption, and prevention of chronic postsurgical pain for patients undergoing breast surgery; however, results vary substantially across studies and there is hardly any data available on the use of TPVB as the sole method of anesthesia for performing breast surgery while patients are awake [22].

We did observe some minor and one major complication. One patient developed severe nausea and vomiting after the epidural application of sufentanil. Close proximity to the area postrema of the brainstem may be a possible cause for this [23]. In one patient the epidural space could not be identified and the procedure was aborted. In one patient the dura was accidentally punctured at the C7/T1 level. Although an unfortunate and stressful event for the anesthetist, no neurological sequelae or other complications were observed. With just one millimeter the epidural space in the cervical region is significantly narrower as compared with the thoracic (4-5 mm) or lumbar (5-6 mm) region. However, since most of the available data being case reports there is a paucity of studies systematically investigating the use of cervical epidural injections and catheters at the cervical level. Some data is available from the field of interventional pain medicine where fluoroscopically guided (cervical) epidural injections are used for managing chronic spinal pain [24]. Although a higher rate of local hematoma was observed as compared to thoracic or lumbar epidurals, no major complications were reported. Injuries to the dural sac were not separately reported. A possible approach to increasing safety of cervical injections may be a prepuncture anatomy check using ultrasound or live puncturing under ultrasound guidance. A study by Kim et al. showed that a prepuncture anatomy check using ultrasound may provide valuable information about skin-space distance and potentially lead to increased safety [25].

Our study has limitations. Firstly, limitations of the retrospective nature of the data apply. Secondly, although this is currently the biggest study investigating cervical epidural blocks in patients undergoing breast cancer surgery, the number of patients is still too small to reliably assess safety. Larger prospective trials and data acquisition through network databases are necessary. Thirdly, we did not investigate the effects of varying local anesthetics or other supplements.

It is conceivable that once these techniques are employed more liberally we will see a significant increase in major complications. Therefore, a systematic in-hospital approach to detecting complications is highly recommended and agreements between anesthesia, neurosurgery, radiology, and neurology should be in place to provide sufficient emergency services in case of catastrophic sequelae. Furthermore, all data derived from these patients should be added to a national or international regional anesthesia network database to ensure that we as anesthesiologists get a better understanding of what risks these techniques may hold once they are employed more liberally.

## 5. Conclusions

Based on our data and previously published data from the field of pain medicine, epidural punctures in the cervical region are feasible methods with a failure rate comparable to thoracic or lumbar epidural punctures. Based on the available data they appear to be relatively safe since major complications such as nerve or spinal cord damage are rarely reported events. However, these are rarely performed techniques and it remains questionable whether safety concerns will change once these techniques are employed more liberally on a wider basis by heterogeneous operators. Data from prospective trials and regional anesthesia network databases are necessary.

Nevertheless, in the light of the current evidence regarding the effects of regional block on cancer recurrence anesthesiologists should be concerned with high epidural and other neuraxial techniques. It may still be too early to tell but if there is any relevant effect on cancer recurrence these anesthetic techniques will become the gold standard and it may become difficult to argue why these techniques should not be employed.

## Figures and Tables

**Table 1:** Demographic data.

Age (yrs)	71.5 ± 16.0
Weight (kg)	71.8 ± 12.8
Height (cm)	165 ± 6
BMI (kg/m^2^)	25.0 ± 7.5
Mastectomy (number of patients)	24
Breast conserving surgery (number of patients)	12
Secondary breast reconstruction (number of patients)	3
Number of epidural punctures at level	
C6/C7	27
C7/T1	9
T1/T2	3
Invasive blood pressure monitoring (number of patients)	15
